# Pulmonary Embolism and Pregnancy—Challenges in Diagnostic and Therapeutic Decisions in High-Risk Patients

**DOI:** 10.3389/fcvm.2022.856594

**Published:** 2022-03-08

**Authors:** Lukas Hobohm, Ioannis T. Farmakis, Thomas Münzel, Stavros Konstantinides, Karsten Keller

**Affiliations:** ^1^Department of Cardiology, University Medical Center of the Johannes Gutenberg-University, Mainz, Germany; ^2^Center for Thrombosis and Hemostasis (CTH), University Medical Center of the Johannes Gutenberg University, Mainz, Germany; ^3^German Center for Cardiovascular Research (DZHK), Partner Site Rhine Main, Mainz, Germany; ^4^Department of Cardiology, Democritus University of Thrace, Komotini, Greece; ^5^Medical Clinic VII, University Hospital Heidelberg, Heidelberg, Germany

**Keywords:** pulmonary embolism, pregnancy, thrombolysis, outcome, venous thromboembolism

## Abstract

Diagnosis of acute PE in pregnant women with haemodynamic instability is following the general integrated risk-adapted diagnostic algorithm and starts with bedside echocardiography to assess RV function. If RV dysfunction is identified, a prompt and immediate reperfusion without further imaging should be initiated. Although pregnancy is listed as a relative contraindication of systemic thrombolysis, in pregnant women with acute PE and haemodynamic instability thrombolysis must be considered. In those cases, other treatment strategies as surgical embolectomy or catheter-directed low-dose thromboylysis or percutaneous thrombectomy should be taken into consideration as well. A multidisciplinary team with experience of PE management in pregnancy should be consulted to reach consensus on the best treatment approach.

## Introduction

Venous thromboembolism (VTE) is considered globally as the third most frequent acute cardiovascular syndrome and is an umbrella term for the clinical entities of acute pulmonary embolism (PE) and deep vein thrombosis (DVT) (1). For PE, annual incidence rates range from 39 to 115 per 100,000 population; for DVT, annual incidence rates between 53 and 162 per 100,000 population were reported (2, 3).

Although an overall decreasing trend in PE-related mortality over the past two decades was observed in a recent analysis of vital registration data in Europe, more than 1% of all deaths in women aged 15–50 years are caused by PE (3, 4). VTE occurs and complicates one of 500–3,000 pregnancies and acute PE is still one of the leading causes of maternal death, also in high-income countries with highly developed medical health services (5, 6). Data from the UK and Ireland demonstrated that thrombosis and thromboembolism were the most common causes of direct maternal death in the years 2013–2015 resulting in 1.13 deaths per 100,000 maternities (7). Additionally, based on current epidemiological data from Germany, PE-related deaths in hospitalized women accounted for almost 14% of all maternal deaths (8).

The management of acute PE during pregnancy is challenging since:

- symptoms of PE (particularly dyspnoea) as well as DVT (especially leg swelling) in pregnant women can in part be difficult to distinguish from “physiological” symptoms of pregnancy,
- lower threshold of PE suspicion,
- fewer publications on validation of PE diagnostic algorithms,
- potential concerns regarding the harm of radiations or iodine contrast exposure regarding PE diagnostics and
- lack of direct evidence from interventional trials regarding PE reperfusion treatment, notably systemic thrombolysis, surgical embolectomy or catheter-directed treatment options (9–11).

Initial risk stratification is based on assessment of the patient's vital/haemodynamic parameters. In haemodynamically stable patients, significant progress has been made in the validation of clinical and biochemical criteria, which are generally considered to apply to pregnant patients as well (7). In contrast, haemodynamic instability in acute PE indicates a high risk of early death and, therefore, rapid reperfusion treatment is recommended, which can however be challenging due to a high risk of bleeding complications in pregnant women.

Aim of this review is to provide a framework for the management of pregnancy- associated PE, especially focusing on critically ill patients.

## Diagnostic Strategies in All patients Vs. Pregnant Women With Suspected PE in the 2019 ESC Guidelines

The diagnostic management of PE in pregnancy is particularly challenging due to the fact that pregnant women often have clinical symptoms, such as shortness of breath or tachycardia, which could point to the suspicion of PE, but can also be present as physiological changes during pregnancy (12). Moreover, overlooking and missing a PE diagnosis could have fatal consequences for mother and child (8), while, on the other hand, thoughtless use of imaging tests could lead to harmful radiation to both mother and fetus (13).

All patients with suspected PE and signs of haemodynamic compromise have a high-risk of death during the first hours and days (14). Thus, initiation of heparin anticoagulation is recommended without delay in patients with high or intermediate clinical probability of PE, while diagnostic workup is in progress (7). The recent published European Society of Cardiology (ESC) guidelines for the diagnosis and management of acute PE underline the importance of a bedside transthoracic echocardiography (TTE) examination in patients with haemodynamic instability. Acute right ventricular (RV) dysfunction can rapidly be detected by TTE if acute PE is the cause of patient's haemodynamic deterioration. If no signs of RV dysfunction exist, other causes of haemodynamic deterioration such as cardiac tamponade, acute coronary syndrome, aortic dissection, acute valvular dysfunction and/or hypovolaemia could be assessed by TTE as well. Additionally, bedside compression ultrasound (CUS) can be used as a further radiation-free diagnostic approach to detect or exclude proximal DVT. If PE is (in)directly confirmed, in all PE patients with haemodynamic instability a rescue thrombolytic treatment is recommended, if no absolute contraindications for systemic thrombolysis are present (7). If these do exist, alternative treatment strategies such as (percutaneous) thrombectomy should be considered. However, there are occasions as haemodynamic collapse with concomitant cardiac arrest and the necessity of cardio-pulmonary resuscitation (CPR) given very limited treatment options. Even if pregnancy is listed as a relative contraindication for systemic thrombolysis, guidelines recommend to consider thrombolysis or surgical embolectomy as the first reperfusion option in these patient group (7, 15). Recent data demonstrated that one third of haemodynamically unstable pregnant women with PE received systemic thrombolytic treatment (8).

**Figure 1:**
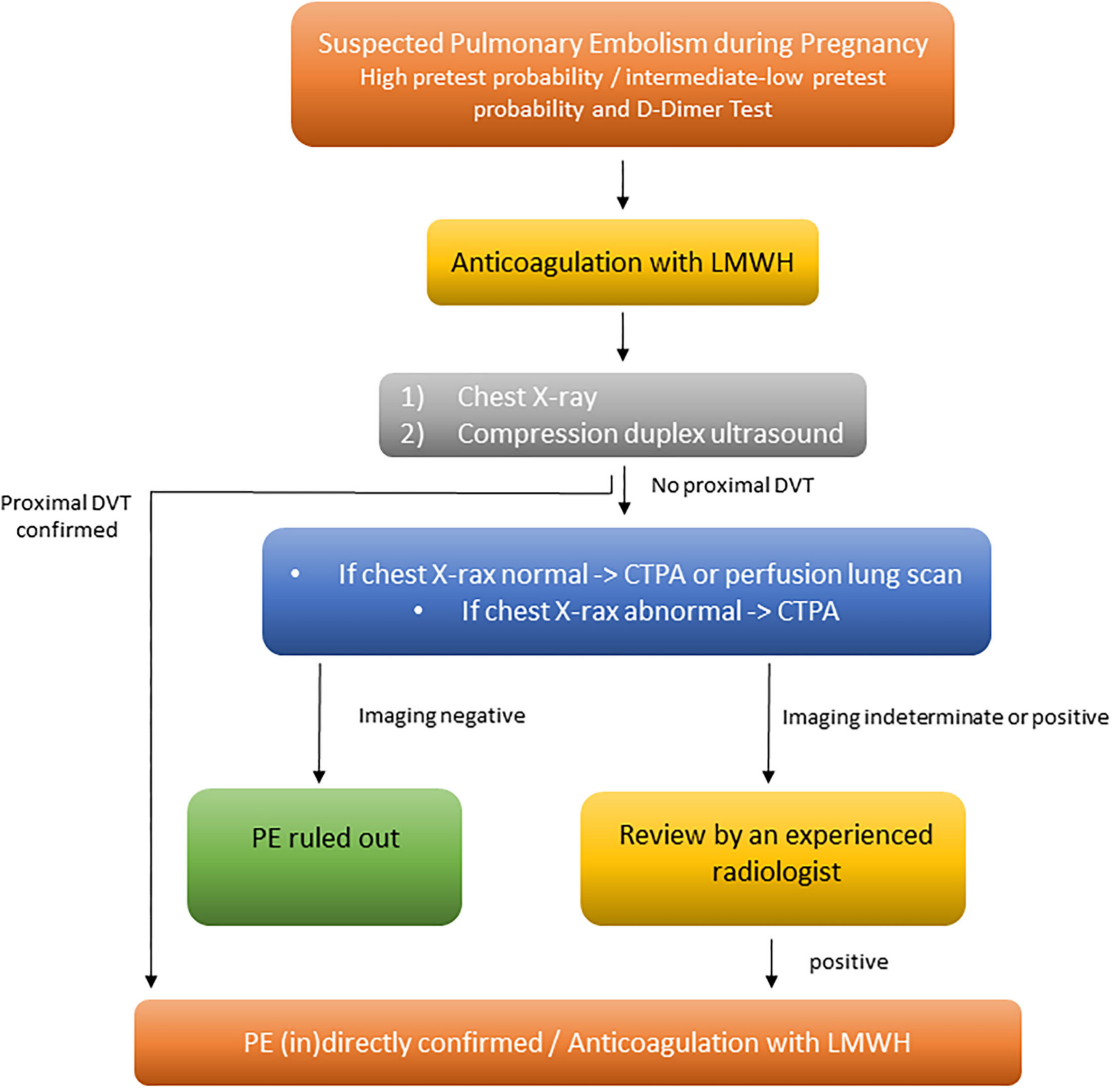
Diagnosis and management of women with suspected acute PE, modified from Konstantinides et al. (7). CTPA, computed tomography pulmonary angiography; DVT, deep vein thrombosis; LMWH, low-molecular-weight heparin; PE, pulmonary embolism.

In contrast to pregnant women with haemodynamic instability, the diagnostic algorithm for normotensive pregnant women may occasionally vary from that used for patients without pregnancy. A pre-test clinical probability assessment along with high-sensitivity D-dimer testing as well as bilateral lower limb CUS are in the center of the diagnostic algorithm for normotensive pregnant women with suspected PE. If there is a high or intermediate pre-test probability, empirical heparin anticoagulation should be administered before diagnostic imaging is initiated (Figure 1). If there are signs/symptoms of DVT, CUS should be performed. If CUS identifies DVT, the diagnosis of PE is—per definition—confirmed indirectly. If no proximal DVT is present or the CUS is inconclusive, chest X-ray followed (in the absence of parenchymal pulmonary changes) by ventilation/perfusion scintigraphy (V/Q scan), or computed tomography pulmonary angiography (CTPA), should be considered to rule out suspected PE (Figure 1).

The overall prevalence of confirmed PE among women is low (2 to 7%) and underlines the diagnostic challenges (16–18). Because of this, and due to the weak level of evidence, current guidelines vary in their approach to diagnosing PE in pregnancy (19). However, recently, two prospective studies have investigated a diagnostic algorithm in women with suspected PE during pregnancy (9, 10). A multicentre prospective diagnostic management study validated the combination of pre-test clinical probability assessment based on the Geneva score, high-sensitivity D-dimer testing, CUS and CTPA in a diagnostic strategy for pregnant women with suspected PE (10). With a low or intermediate pre-test clinical probability and a negative D-dimer result, PE was excluded. All other patients underwent lower limb CUS and, if results were negative, CTPA was performed. In total, 395 women were included and among these, PE was diagnosed in 28 (7.1%) and excluded in 367 (92.9%). The rate of symptomatic venous thromboembolic events was 0.0% (95% CI, 0.0 to 1.0%) among untreated pregnant women after exclusion of PE on the basis of negative results on the diagnostic work-up. Therefore, this diagnostic algorithm involving sequential assessment of pre-test clinical probability based on the Geneva score, D-dimer measurement, lower limb CUS and CTPA or V/Q scan is able to safely rule out PE in pregnancy (10). Another prospective study involving pregnant women with suspected PE assessed three criteria from the so-called YEARS algorithm (clinical signs of DVT, haemoptysis, and PE as the most likely diagnosis), also taking the D-dimer levels into account. A total of 498 women were included in this study and of these, PE was diagnosed in 20 (4.0%) of the examined patients and excluded in 478 (96%) women.

The current ESC guidelines recommend to perform an X-ray in pregnant women with suspected PE. If the X-ray is normal, V/Q scan should be performed, due to the fact, that V/Q scan is associated with low fetal and maternal radiation exposure. If the X-ray is abnormal, showing, for example, pulmonary infiltrates, then CTPA should be performed directly (7, 17) (Figure 1).

## Diagnostic Strategies Across Guidelines and Societies in Pregnant Women With Suspected High-Risk PE

International medical society guidelines address new evidence of diagnostic strategies in pregnant women with suspected PE (7, 20–25). In line, to the aforementioned 2019 ESC guidelines, the American Thoracic and Radiology Society (ATS-STR), Society of Thrombosis and Haemostasis (GTH) and Royal College of Obstetricians and Gynaecologists (RCOG) guidelines begin with administering empirical therapeutic anticoagulation, if haemodynamic instability is present, even before any diagnostic work-up is started. The RCOG (24) and ESC (7) guidelines recommend early treatment for all patients suspected of PE with high- or intermediate clinical probability, while diagnostic workup is in progress. GTH (23) and ATS-STR (21) guidelines recommend empirical treatment in patients with a high clinical probability of having PE only (26). The remaining guidelines of Australasian Society of Thrombosis and Haemostasis and the Society of Obstetric Medicine of Australia and New Zealand (ASTH-SOMANZ), European Association of Nuclear Medicine (EANM), and Society of Obstetricians and Gynaecologists of Canada (SOGC) do not mention any empirical treatment (20, 22, 25). The ESC guidelines, as the only one, recommend the use of echocardiography as a first risk assessment strategy in all patients with haemodynamic instability (7).

## Treatment of Acute Pulmonary Embolism in Pregnant Women—High-Risk vs. Not High-Risk

Especially high-risk PE in pregnancy can be a devastating event with a high case-fatality rate up to 37% (8). In patients with haemodynamic instability, unfractionated heparin (UFH) is used as a first-line medication. If the haemodynamic status aggravates, thrombolytic agents may be necessary to administer. Immediate thrombolytic treatment is recommended unless absolute contraindications for systemic thrombolysis are present (7). Besides thrombolysis, other treatment options of high-risk PE as surgical or percutaneous thrombectomy in should be taken into account. If necessary also extra-corporeal membrane oxygenation (ECMO) can be considered for depressurize the right ventricle and pulmonary circulation (27). Although pregnancy is reported as a relative contraindication of thrombolysis, haemodynamic collapse with concomitant cardiac arrest and the necessity of CPR leave the clinician with limited alternative treatment options (7). Recent data demonstrated that one third of unstable women with PE receive systemic thrombolytic treatment (8). Thrombolysis might be associated with a favorable outcome (94 and 88% of maternal and fetal survival, respectively) (27). However, other data of retrospective nature provide a more ominous prognostic depiction of thrombolysis in the context of high-risk PE. A mortality rate of 42.6% were reported among 67 pregnant women who received thrombolysis (8). Furthermore, in the same study, thrombolysis was sparsely used and regarded as a last resort option; even in the presence of haemodynamic collapse, only 37.8% of patients received thrombolysis.

Bleeding complications are reported as a common adverse event after thrombolytic treatment in 18 to 58% cases during pregnancy and in the post-partum period, respectively (27). Maternal major bleeding was reported in 3 out of 10 cases. Most of them were vaginal or abdominal C-section associated occurring in the early post-partum period. Especially the peripartum phase as well as spinal or epidural anesthesia are associated with high risk of bleeding (7). Therefore, thrombolytic therapy should be used peripartum in a life-threatening context only. The risk for the fetus is low, because a transplacental crossing of fibrinolytic drugs is very unlikely due to the fact that their components are larger than 1,000 Dalton (28, 29). However, the lack of prospectively designed controlled studies precludes conclusions regarding the efficacy and safety profile of thrombolysis in high-risk pregnancy-associated PE. Thus, causalities of fatal maternal and fetal outcomes cannot be deduced to the administration of the thrombolytic agent only.

In the case of absolute contraindications, alternative treatment strategies such as surgical embolectomy or percutaneous low-dose thrombolysis (CDT) or thrombectomy should be considered (30) (Table 1). Results of several studies confirm that CDT, a novel treatment modality for high- and intermediate high-risk PE, is associated with a favorable outcome regarding bleeding complications in comparison to systemic thrombolysis in patients with PE (31). However, randomized studies using standardized clinical outcomes such as mortality and recurrent VTE are missing. In order to close this gap, CDT is currently being evaluated in a phase III clinical trial (NCT04790370). However, pregnancy constitutes an exclusion criterion of the trial and only few cases of pregnant women treated with CDT have been published in literature yet (27, 45, 46). Surgical embolectomy or percutaneous thrombectomy are reasonable treatment options, when needed in the immediate postpartum period, to avoid the bleeding risks of thrombolysis. However, these methods are limited in their availability and are used as last life-saving therapy option only (27). However, if reperfusion treatment is not effective or not available in the setting of haemodynamic instability, data indicate that the temporary use of mechanical circulatory support via ECMO as a bridging therapy might improve outcomes until pharmacological or mechanical thrombolysis or embolectomy is applied (47). In patients with acute PE and pregnancy ECMO has not been widely used. In a systematic review of 21 pregnant women with PE and ECMO support, the maternal survival rate was 76%, while the fetal survival rate was 63% (48).

**Table 1:** Therapeutic strategies for catheter-directed treatment adapted from Hobohm et al. (31).

**Technique**	**Description**	**Device (company)**	**Evidence**
Catheter-directed thrombolysis	The catheter is inserted directly into the pulmonary artery and the thrombolytic agent released close to the location of the thrombus occlusion.	Cragg-McNamara® (Ev3 Endovascular); UniFuse® (AngioDynamics): Multi-sidehole pigtail catheter with 4–5 French	Observational studies and one randomized trial (31–33)
Ultrasound-assisted catheter-directed thrombolysis	A second catheter lumen contains low-energy ultrasound transducers which should loosen the clot structure to facilitate thrombolytic penetration.	EkoSonic® (BTG) 5.2 French device	Prospective, single group studies and prospective randomized trials (34–36)
Catheter-directed embolectomy by fragmentation	The pigtail is inserted into the distal part of the thrombus and rotating while retracting at the proximal part.	Pigtail 5 French fragmentation plus thrombectomy with Aspirex® 8/10 French	Observational studies (37, 38)
Catheter-directed embolectomy, rheolytic	High-pressure jet streams disrupt the thrombus, which is then trapped in a low-pressure zone and aspirated in the catheter.	AngioJet® (Boston Scientifics) 6 French catheter	Observational studies (39, 40)
Catheter-directed embolectomy by suction	The thrombus is aspirated via a pump, reintroducing excess aspirated blood via a veno-venous bypass system or with mechanical clot engagement.	AngioVac® (AngioDynamics) suction cannula with 26 French access; Indigo (Penumbra) 8 French vacuum-assisted aspiration system	Observational studies (41, 42)
Catheter-directed embolectomy by entrapment	Self-expanding nitinol disks are placed into the thrombus, ensnare it by expanding, and are retracted into the catheter.	FlowTriever® (Inari) 20 French device	Observational studies and one single-arm phase II trial (43, 44)

An additional treatment option for pregnant women with absolute contraindications for anticoagulation could be the placement of an inferior vena cava (IVC) filter (7). Data on this preventive approach is limited. A systematic review including 124 pregnant women with DVT, in whom an IVC filter was inserted, were analyzed. No fatal PE occurred after filter placement and retrieval complication rates appeared comparable to those in the general population (49). However, even if the authors concluded that IVC filters can be used effectively in pregnancy to prevent PE, there is currently not enough evidence to suggest that IVC filters should be used routinely (50–52). In exceptional cases with absolute contraindications for anticoagulation, or if recurrent PE is present despite adequate therapeutic anticoagulation, IVC should be taken into consideration (7). Overall, the evidence for advanced treatment options in high-risk PE during pregnancy is poor. A prospective international registry investigating the effectiveness and safety of advanced methods in massive pregnancy-related PE is currently underway (MAPP registry endorsed by the International Society on Thrombosis and Haemostasis) (53). Due to the diagnostic and treatment complexity, a multidisciplinary team (with experience in PE management in pregnancy) should be consulted to evaluate the best and treatment approach (7).

Anticoagulation remains the mainstay of treatment in pregnancy and must be administered to all patients with high-risk suspicion of PE and confirmed PE (7). Since heparins do not pass the placenta and are not associated with teratogen effects on the fetus, they can be safely administered in pregnant women. Low molecular weight heparins (LMWH) are the agents of choice, because they have a predictable pharmacodynamic profile (54). In contrast, vitamin K antagonists (VKAs) can cause teratogenicity and fetal bleeding during the first and the third trimester and should therefore not used during those periods (55). Due to the insufficient safety data, direct oral anticoagulants (DOACs) are also contraindicated during pregnancy (56, 57). UFH may be associated with heparin-induced thrombocytopenia, resulting in restriction of recommendation regarding their use. However, in pregnant women heparin-induced thrombocytopenia is extremely rare (<0.1%) (58). UFH is used predominantly for patients with severe renal impairment, extreme body weight, high-risk PE, and PE occurring very close to delivery (59). Dosing strategies of LMWH generally follow these of the non-pregnant population, as there is a lack of specific randomized data (60). Although evidence suggest that most anticoagulated patients lie in a sub-therapeutic range, anti-Xa level monitoring has not be shown to be beneficial. LMWH use is currently recommend only for patients with severe renal impairment and extremes of body weight (61–63). However, therapeutic use of LMWH or UFH has a 3 and 2% incidence risk for antepartum and postpartum hemorrhagic complications, respectively (64). Approaching delivery, LMWH is usually converted to a continuous UFH infusion ≥36 h prior to delivery, especially if neuraxial anesthesia is planned. Finally, UFH should be paused 4–6 h prior to delivery. The timeframe of the post-partum re-initiation of LMWH should be decided by a multidisciplinary team and depends on the mode of delivery as well as the thrombotic and bleeding risk profile of the patient. Importantly, re-initiation of LMWH should not start 4 h after the epidural catheter has been removed (7). If there is an allergy or adverse response to LMWH, Fondaparinux is given as an alternative drug, although solid data are lacking and minor transplacental passage has been demonstrated (65).

## Conclusion

Diagnosis of acute PE in pregnant women with haemodynamic instability

- is following the general integrated risk-adapted diagnostic PE algorithm PE and
- starts with bedside echocardiography to assess RV function. If RV dysfunction is identified, a prompt and immediate reperfusion without further imaging should be initiated.

Although pregnancy is listed as a relative contraindication of systemic thrombolysis, in pregnant women with acute PE and haemodynamic instability

- systemic thrombolysis must be considered and
- other treatment strategies as surgical embolectomy or catheter-directed low-dose thromboylysis or percutaneous thrombectomy should be taken into consideration as well.

A multidisciplinary team with experience of PE management in pregnancy should be consulted to reach consensus on the best treatment approach.

## Author Contributions

All authors: conception and design of the study, data collection, analysis of the data, interpretation of data, drafting of the manuscript and revising of the manuscript critically for important intellectual content, and final approval of the manuscript submitted.

## Conflict of Interest

LH reports lecture/consultant fees from MSD and Janssen, outside the submitted work. SK reports institutional grants and personal lecture/consultant fees from Bayer AG, Daiichi-Sankyo, and Boston Scientific; institutional grants from Inari Medical; and personal lecture/consultant fees from Pfizer- Bristol-Myers Squibb and MSD, all outside the submitted work.

## Publisher's Note

All claims expressed in this article are solely those of the authors and do not necessarily represent those of their affiliated organizations, or those of the publisher, the editors and the reviewers. Any product that may be evaluated in this article, or claim that may be made by its manufacturer, is not guaranteed or endorsed by the publisher.
